# Downregulation of ROR2 attenuates LPS‐induced A549 cell injury through JNK and ERK signaling pathways

**DOI:** 10.1002/iid3.803

**Published:** 2023-04-26

**Authors:** Zhonglin Wang, Liu Yang

**Affiliations:** ^1^ Department of Anesthesiology Yongchuan Hospital Affiliated to Chongqing Medical University Yongchuan People's Republic of China

**Keywords:** acute lung injury, ERK, JNK, ROR2, small interfering RNA

## Abstract

**Background:**

We aimed to determine whether receptor tyrosine kinase‐like orphan receptor 2 (ROR2) is involved in the occurrence of acute lung injury (ALI) by an animal study and explore the effect of ROR2 downregulation on lipopolysaccharide (LPS)‐treated human lung carcinoma A549 cells by a cytological study.

**Methods:**

Murine models of ALI were successfully constructed by intratracheal instillation of LPS. Meanwhile, A549 cell line stimulated with LPS was used for a cytological study. The expression of ROR2 and its effect on proliferation, cell cycle, apoptosis, and inflammation were detected.

**Results:**

It was found that LPS administration markedly inhibited the cell proliferation, resulted in cell cycle arrest at G1 phage, elevated levels of pro‐inflammatory cytokines and apoptosis rate of A549 cells. However, LPS‐mediated adverse effects mentioned above were significantly ameliorated by downregulation of ROR2 in comparison with LPS treatment. In addition, administration of ROR2 siRNA notably decreased the phosphorylation level of c‐Jun N‐terminal kinase (JNK) and extracellular signal‐regulated kinase (ERK) in LPS‐challenged A549 cells.

**Conclusions:**

Thus, the present data indicate that downregulation of ROR2 may decrease LPS‐induced inflammatory responses and cell apoptosis through inhibiting JNK and ERK signaling pathway, which attenuates ALI.

## Graphical abstract

The present data indicate that downregulation of receptor tyrosine kinase‐like orphan receptor 2 may decrease lipopolysaccharide‐induced inflammatory responses and cell apoptosis through inhibiting of c‐Jun N‐terminal kinase and extracellular signal‐regulated kinase signaling pathway, which attenuates acute lung injury.

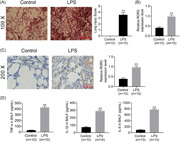

## 1 INTRODUCTION

Acute respiratory distress syndrome (ARDS) is a common and devastating clinical illness characterized by impaired oxygenation (refractory hypoxemia), pulmonary edema, decreased lung compliance, bilateral diffuse pulmonary infiltration, the loss of alveolar barrier function, and massive pulmonary and systemic release of pro‐inflammatory mediators.^1^, ^2^ Acute lung injury (ALI), considered as the early clinical manifestation of ARDS, results from direct and indirect pulmonary insults.^3^

The excessive release of endotoxin‐induced inflammatory mediators is one of the leading causes of ALI.^4^, ^5^, ^6^ Since sepsis is considered to be the major risk factor for pathogenesis and development of ALI, the sepsis‐associated ALI animal model is often established by the intratracheal administration of lipopolysaccharide (LPS), the major endotoxin of gram‐negative bacteria. As yet, no effective pharmaceutical therapies have been developed for ALI. It is well known that RNA‐based molecular targeting treatment is a potential therapeutic approach for stubborn and incurable diseases.

Receptor tyrosine kinase‐like orphan receptor 2 (ROR2), encoding an orphan receptor tyrosine kinase, is selectively expressed in specific cell lineages, which plays critical roles in regulating cell cycle, migration, proliferation, and apoptosis as well as inflammatory responses by initiating signaling cascades.^7^, ^8^ ROR2 is an important receptor involved in the Wnt5a‐mediated inflammatory response in atherosclerosis^9^ and PM2.5‐related chronic obstructive pulmonary disease^10^ as well as the malignancies.^11^ Extracellular signal‐regulated kinase (ERK) and c‐Jun N‐terminal kinase (JNK) are important regulatory proteins in the inflammatory responses of LPS‐induced ALI.^12^, ^13^ The aim of the present study was to determine whether ROR2 is involved in the pathogenesis and development of ALI by an animal study and explore the effect of ROR2 downregulation on LPS‐treated human lung carcinoma A549 cells by a cytological study.

## 2 MATERIALS AND METHODS

### 2.1 Animal grouping and treatment

A total of 20 male wild‐type C57BL/6 mice (age, 8–10 weeks) were obtained from the Experimental Center of China Second Military Medical University and randomly allocated into one of two groups (*n* = 10): control group and LPS‐induced ALI model group.

Murine model of ALI was established by intratracheal instillation of LPS, as previously described.^10^, ^11^ Briefly, the mice were weighted and anesthetized with 5% chloral hydrate (intraperitoneally, 100 mg/kg). The cervical region was shaved and disinfected with 75% alcohol. After careful dissection, the trachea was exposed. LPS (from *Escherichia coli* 055:B5; L2880; Sigma‐Aldrich; 15 mg/kg) was subsequently slowly injected into trachea of the mice. Following LPS administration for 12 h, mice were euthanized through cervical dislocation, and Lung tissue samples were then harvested for conventional hematoxylin and eosin (H&E) and immunohistochemical staining. Bronchoalveolar lavage fluid (BALF) were collected and centrifuged at 4°C, and the supernatants were analyzed by enzyme‐linked immunosorbent assay (ELISA).

### 2.2 Immunohistochemistry (IHC)

The IHC staining was performed on formalin‐fixed, paraffin‐embedded sections. Slides (5 μm) were treated with 0.3% hydrogen peroxide for 30 min, followed by incubation overnight with anti‐ROR2 (Abcam) using a 1:100 working dilution. The sections were visualized using diaminobenzidine for 10 min, counterstained with hematoxylin, and examined by microscopy.

### 2.3 Quantitative polymerase chain reaction (qPCR) analysis

Total RNA of lung tissues was extracted by Trizol (Invitrogen), and cDNA was synthesized using First Strand cDNA Synthesis Kit (Thermo Scientific). The qPCR was performed using a 40‐cycle two‐step PCR with sequence‐specific primer pairs on the ABI 7900 fast real‐time detection system (Invitrogen). Primers were designed using Primer 5.0 software and the sequences of ROR2 were as follows: forward, 5′‐GTCCAACGCACAGCCCAAATC‐3′ and reverse, 5′‐CCGGTTGCCAATGAAGCGTG‐3′. The primer sequences of glyceraldehyde phosphate dehydrogenase (GAPDH) as the housekeeping control was forward, 5′‐GGAGCGAGATCCCTCCAAAAT‐3′ and reverse, 5′‐GGCTGTTGTCATACTTCTCATGG‐3′.

### 2.4 Cell culture and transfection

Human alveolar epithelial A549 cells (ATCC® Number: CRM‐CCL‐185™) were purchased from Institute of Shanghai Biochemistry and Cell Biology, Chinese Academy of Sciences. A549 cells were cultured in Roswell Park Memorial Institute 1640 medium supplemented with 5% fetal bovine serum (FBS; Sijiqing Biological Engineering Material Co., Ltd.) according to the instructions of American Type Culture Collection (ATCC). A549 cells were grown in six‐well culture plates and allowed to adhere for 12 h at 37°C in a humidified atmosphere with 5% CO_2_. After serum starvation for 18 h, cells were transfected with ROR2‐targeting siRNA (2.9 × 10^8^ plague forming unit/mL) or negative control siRNA. Transfection mixtures were premixed in 250 μL serum‐free medium before addition to the cells. After 48 h of transfection, the cells were then treated with 10 μg/mL LPS for 24 h.

### 2.5 Cell proliferation assay

Cell proliferation assay was performed using cell counting kit‐8 (CCK‐8; Beyotime Biotechnology). In brief, 100 μL cell suspension was prepared in a 96‐well plate at 1 × 10^4^ cells/well, and incubated for 24, 48, or 72 h. Then, 10 μL CCK‐8 solution was added to each well and incubated for 1 h. The absorbance at 450 nm was measured with a microplate analyzer.

### 2.6 Cell cycle and apoptosis analysis

Cell cycle was examined with the Cell Cycle and Apoptosis Analysis Kit (Beyotime Biotechnology), while cell apoptosis was examined with the Annexin V‐FITC Apoptosis Detection Kit (Invitrogen) according to the instructions. After staining, samples were analyzed using flow cytometer (Beckman Coulter). Hoechst 33342 staining was also used to evaluate cell apoptosis as previous described.^14^

### 2.7 ELISA

Culture supernatants were collected and centrifuged at 3000*g* for 15 min. The supernatants were used for ELISA analysis. Measurement of inflammatory cytokines including tumor necrosis factor‐α (TNF‐α), interleukin‐1β (IL‐1β), and IL‐8 in culture supernatants and BALF were performed using commercially available ELISA kits.

### 2.8 Western blot analysis

After exacting from cultured cells using radio immuno precipitation assay (Thermo Scientific), 30 μg protein were subjected to electrophoresis. The protein was transferred to polyvinylidene fluoride (PVDF) membranes at 250 mA at 4°C for 2 h. After incubation with following indicated antibodies overnight at 4°C: ROR2, GAPDH, cell division cycle protein 2 (CDC2), cyclin B1 (CCNB1), caspase‐3, caspase‐9, JNK, p‐JNK, ERK1/2 and p‐ERK1/2 (1:1000 dilution; Cell Signaling Technology), the membranes were washed with tris buffered saline tween (TBST) and stained with secondary antibody (1:5000 dilution) at room temperature for 2 h. After adding the Chemiluminescent Substrates (Invitrogen), the bands were scanned.

### 2.9 Statistical analysis

Values were expressed by the mean ± standard deviation (SD). SPSS 18.0 software (SPSS, Inc.) was used for data analysis using Student's *t* test or analysis of variance with the Dunnett's multiple comparison test. *p* < .05 was regarded as statistically significance.

## 3 RESULTS

### 3.1 ROR2 mRNA and protein are highly expressed in murine models of LPS‐induced ALI

**Figure 1:**
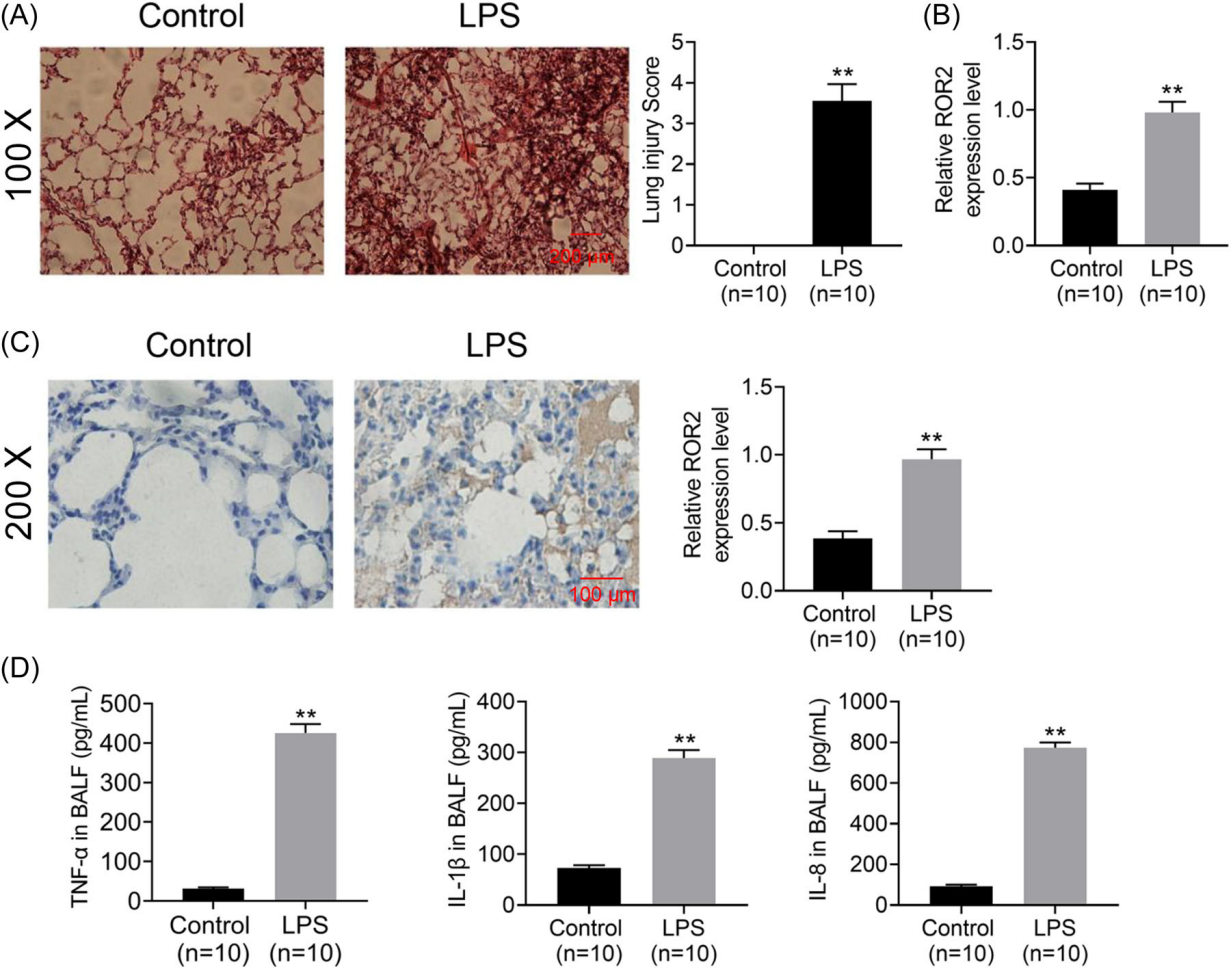
Effect of intratracheal LPS administration on lung of mice (*n* = 10 per group). (A) Histopathological examination of lung tissues in control and LPS‐treated mice stained with H&E and the severity of lung injury evaluated using semiquantitative histological scoring. Scale bar = 200 μm. Magnification: ×100. (B) A qPCR analysis with sequence‐specific primers of ROR2 in the lung tissue of control and LPS‐treated mice. (C) Immunohistochemical analysis of ROR2 protein in the lung tissues of control and LPS‐treated mice and quantitative analysis of ROR2 expression. Scale bar = 100 μm. Magnification: ×200. (D) The contents of TNF‐α, IL‐1β, and IL‐8 in BALF of mice were detected by using a colorimetric sandwich ELISA kit. Data are presented as mean ± standard deviation. SPSS 18.0 was used for data analysis using Student's *t* test or ANOVA with the Dunnett's multiple comparison test. ANOVA, analysis of variance; BALF, bronchoalveolar lavage fluid; ELISA, enzyme‐linked immunosorbent assay; H&E, hematoxylin and eosin; IL‐1β, interleukin‐1β; LPS, lipopolysaccharide; qPCR, quantitative polymerase chain reaction; ROR2, receptor tyrosine kinase‐like orphan receptor 2; TNF‐α, tumor necrosis factor‐α.

To determine whether ROR2 was involved in the pathogenesis and development of ALI, murine models of ALI was constructed by intratracheal administration of LPS; the results of histopathological examination indicate that murine models of LPS‐induced ALI are constructed successfully (Figure 1A). Furthermore, ROR2 mRNA and protein expression in the lung tissues were examined by qPCR with sequence‐specific primers and IHC, respectively. As illustrated in Figure 1B, ROR2 mRNA expression in lung tissues of LPS‐challenged mice was significantly higher than that of the control group. Immunohistochemical staining of ROR2 protein was scarcely detected in the lung tissues of control group, whereas, widespread pale brown staining was observed in the lung tissues of LPS‐treated mice (Figure 1C). Colorimetric sandwich ELISA kit detection of TNF‐α, IL‐1β, and IL‐8 in BALF in LPS‐stimulated mice showed that these inflammatory cytokines were significantly upregulated in BALF (Figure 1D).

### 3.2 Downregulation of ROR2 enhances proliferation, promotes cell cycle process, and inhibits apoptosis of LPS‐treated A549 cells in vitro

**Figure 2:**
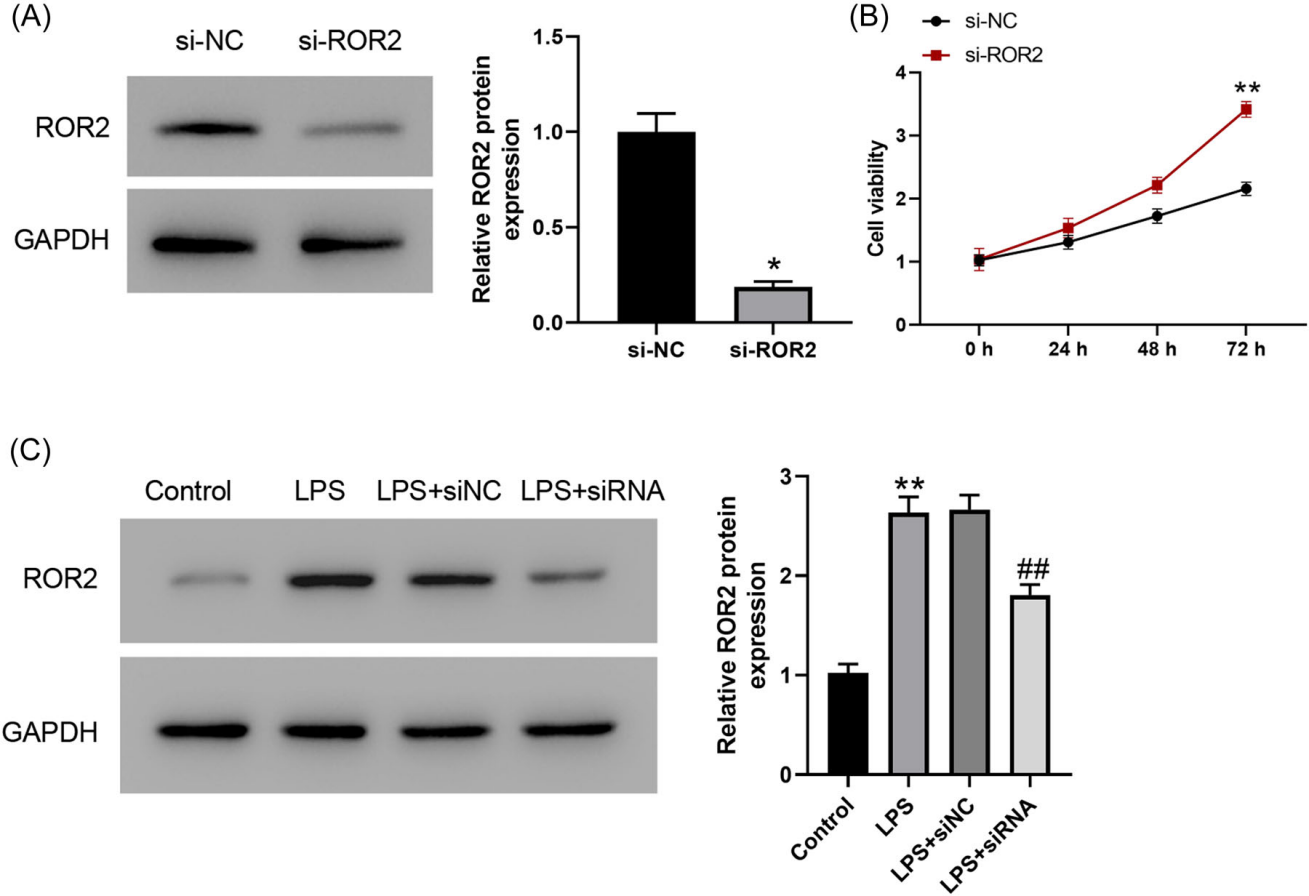
(A) ROR2 knockdown efficiency was measured in A549 cells transfected with siNC or si‐ROR2. (B) The cell proliferation ability was determined in A549 cells transfected with siNC or si‐ROR2. (C) The protein levels of ROR2 were determined in A549 cells with ROR2 knockdown before LPS treatment. Values are expressed as mean ± standard deviation. SPSS 18.0 was used for data analysis using Student's *t* test or ANOVA with the Dunnett's multiple comparison test. *p < .05, **p < .01 compared with control group or siNC group. ^##^ *p* < .01, compared with LPS + siNC group. **p < .01. ANOVA, analysis of variance; LPS, lipopolysaccharide; ROR2, receptor tyrosine kinase‐like orphan receptor 2.

**Figure 3:**
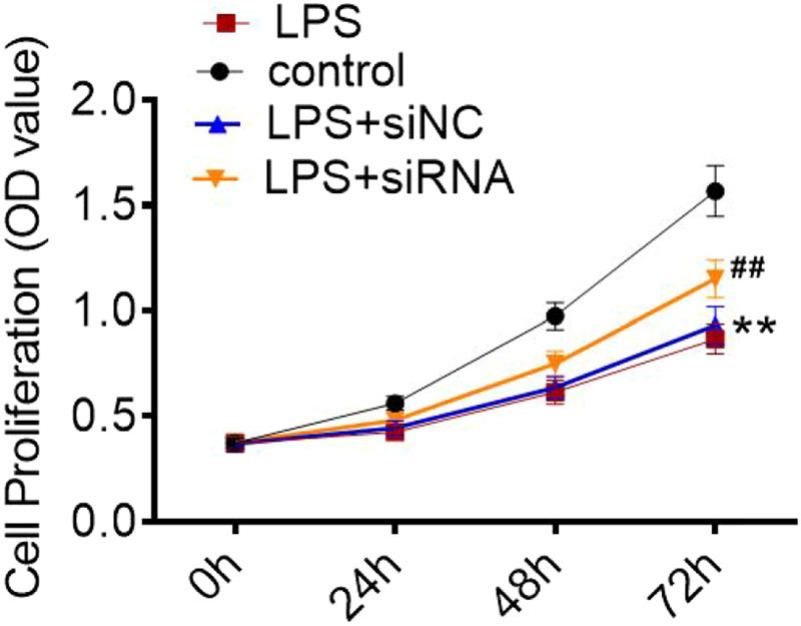
Effect of ROR2 downregulation on proliferation of LPS‐treated A549 cells in vitro. A549 cell proliferation was examined by CCK‐8 method in A549 cells with ROR2 knockdown before LPS treatment. Data are presented as mean ± standard deviation (*n* = 3). SPSS 18.0 was used for data analysis using Student's *t* test or ANOVA with the Dunnett's multiple comparison test. **p < .01, compared with control group; ^##^ *p* < .01, compared with LPS + siNC group. ANOVA, analysis of variance; CCK‐8, cell counting kit‐8; LPS, lipopolysaccharide; ROR2, receptor tyrosine kinase‐like orphan receptor 2.

**Figure 4:**
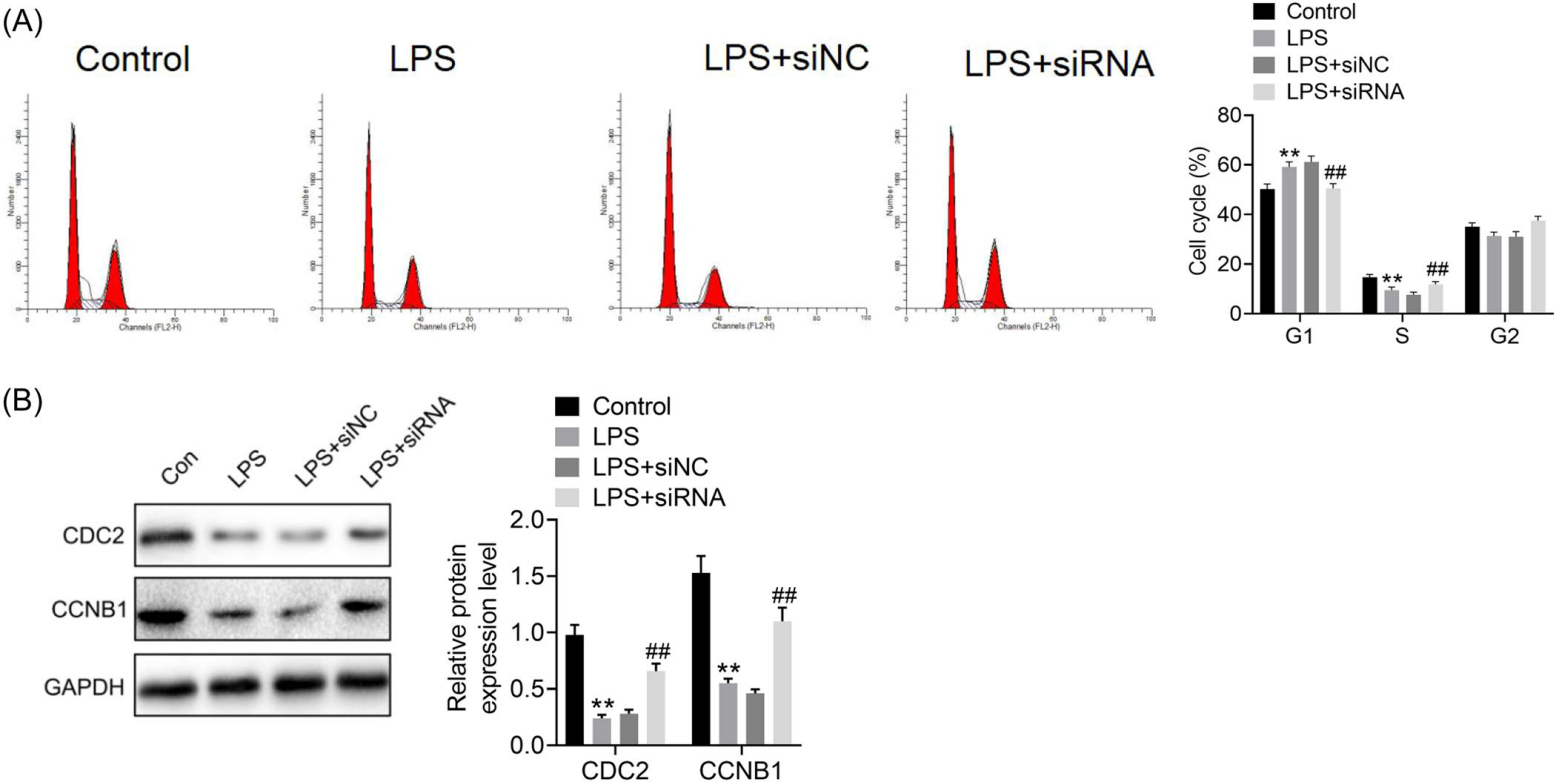
Effect of ROR2 downregulation on cell cycle of LPS‐treated A549 cells in vitro. (A) The cell cycle analysis of A549 cells with ROR2 knockdown before LPS treatment was determined by FCM. (B) The protein levels of cell cycle‐related proteins CDC2 and CCNB1 were determined by western blot analysis. Data are presented as mean ± standard deviation (*n* = 3). SPSS 18.0 was used for data analysis using Student's *t* test or ANOVA with the Dunnett's multiple comparison test. **p < .01 compared with control group; ^##^ *p* < .01 compared with LPS + siNC group. ANOVA, analysis of variance; CCK‐8, cell counting kit‐8; CCNB1, cyclin B1; CDC2, cell division cycle protein 2; LPS, lipopolysaccharide; ROR2, receptor tyrosine kinase‐like orphan receptor 2. FCM, flow cytometry.

**Figure 5:**
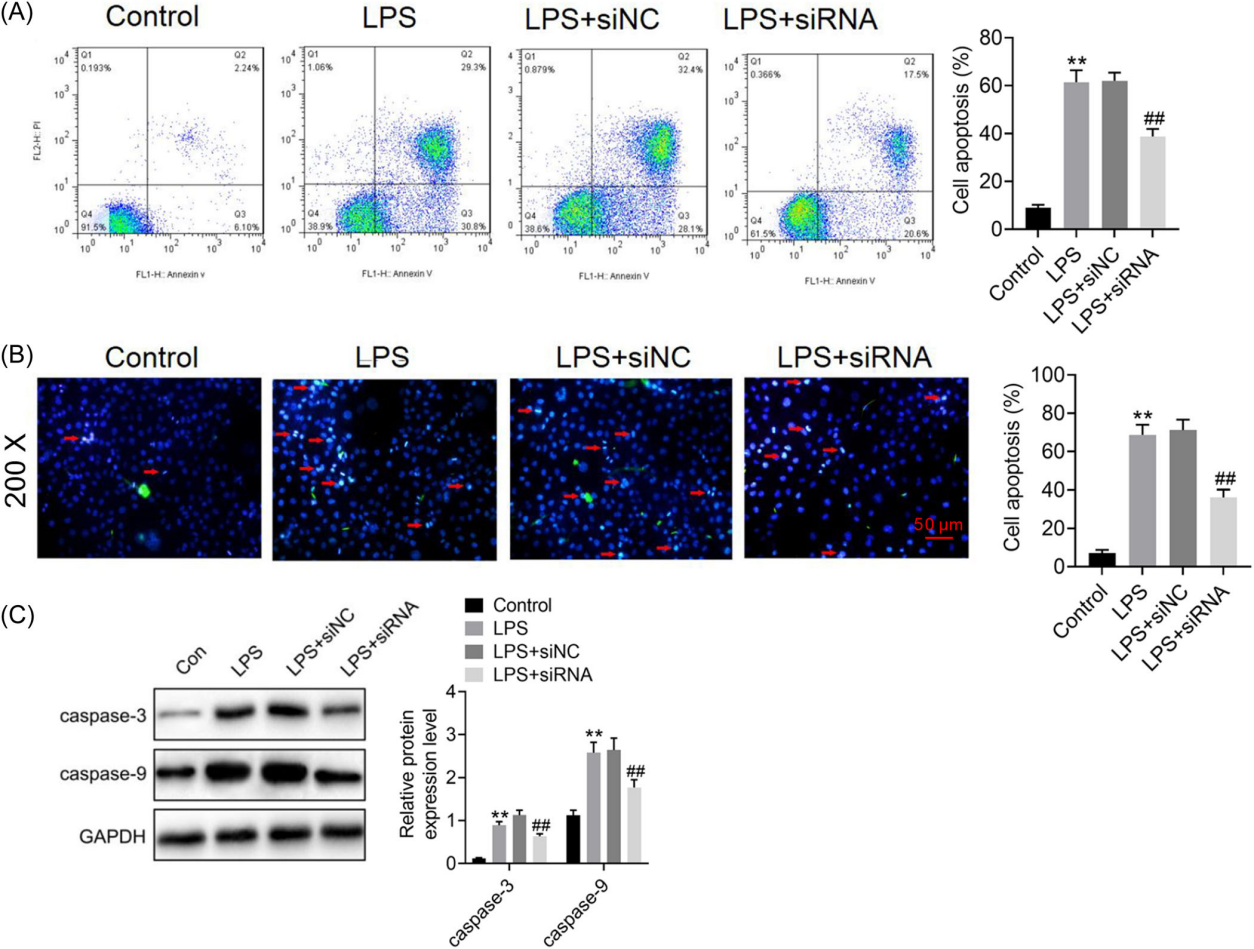
Effect of ROR2 downregulation on apoptosis of LPS‐treated A549 cells in vitro. (A) The cell apoptosis analysis of A549 cells with ROR2 knockdown before LPS treatment was determined by FCM. (B) Hoechst staining was carried out to analyze cell apoptosis. Scale bar = 50 μm. Magnification: ×200. (C) The protein levels of apoptosis‐associated proteins, caspase‐3 and caspase‐9 were determined by western blot analysis. Data are presented as mean ± standard deviation (*n* = 3). SPSS 18.0 was used for data analysis using Student's *t* test or ANOVA with the Dunnett's multiple comparison test. **p < .01 compared with control group; ^##^ *p* < .01 compared with LPS + siNC group. ANOVA, analysis of variance; CCK‐8, cell counting kit‐8; CCNB1, cyclin B1; CDC2, cell division cycle protein 2; LPS, lipopolysaccharide; ROR2, receptor tyrosine kinase‐like orphan receptor 2. FCM, flow cytometry.

To determine the involvement of ROR2 in LPS‐induced A549 cell injury, we first transfected ROR2 siRNA into A549 cells, which was further induced by LPS for 24 h. Western blot analysis revealed that the expression level of ROR2 was dramatically decreased by ROR2 siRNA (Figure 2A). We found that cell proliferation was markedly increased in A549 cells with ROR2 knockdown (Figure 2B). In addition, ROR2 inhibition effectively inhibited LPS‐induced the increased expression of ROR2 (Figure 2C). The effects of ROR2 downregulation on proliferation, cell cycle, and apoptosis of LPS‐treated A549 cells in vitro were further investigated. Compared with control group, LPS administration notably decreased the cell viability; nevertheless, downregulation of ROR2 markedly attenuated the decrease in proliferation of LPS‐challenged A549 cells; there was no significant difference in cell proliferation between LPS treatment and negative control siRNA treatment (Figure 3). LPS treatment notably blocked the entry of A549 cells from G1 phage into S phage of cell cycle; on the contrary, ROR2 downregulation markedly attenuated LPS‐treated A549 cell arrest at G1 phage (Figure 4A). As illustrated in Figure 4B, expression of cell cycle‐related proteins, CDC2 and CCNB1, was significantly decreased by LPS; no statistically significant difference was found between LPS treatment and negative control siRNA treatment; however, ROR2‐targeting siRNA treatment markedly upregulated the expression of CDC2 and CCNB1. Following LPS administration, higher rate of apoptosis was observed in A549 cells, while the increase in the apoptosis rate of LPS‐challenged A549 cells was remarkably attenuated by downregulating ROR2 (Figure 5A,B). In addition, LPS administration significantly increased the expression of caspase‐3 and caspase‐9, and the increase in the expression of caspase‐3 and caspase‐9 was significantly attenuated by downregulation of ROR2 (Figure 5C).

### 3.3 Downregulation of ROR2 inhibits pro‐inflammatory cytokine release from LPS‐treated A549 cells in vitro

**Figure 6:**
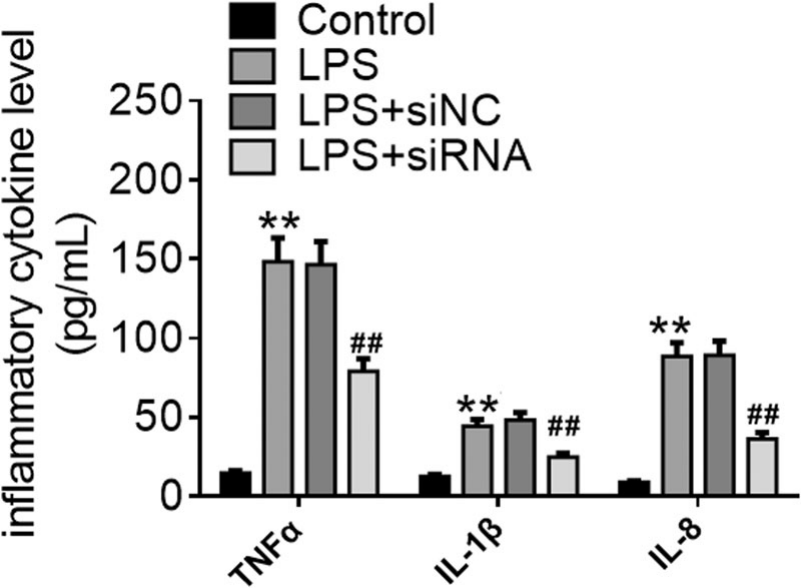
Effect of ROR2 downregulation on pro‐inflammatory cytokine release from LPS‐treated A549 cells in vitro. The contents of TNF‐α, IL‐1β, and IL‐8 in culture supernatant were detected by using a colorimetric sandwich ELISA kit. Data are presented as mean ± standard deviation (*n* = 3). SPSS 18.0 was used for data analysis using Student's *t* test or ANOVA with the Dunnett's multiple comparison test. **p < .01 compared with control group; ^##^ *p* < .01 compared with LPS + siNC group. ANOVA, analysis of variance; ELISA, enzyme‐linked immunosorbent assay; IL‐1β, interleukin‐1β; IL‐8, interleukin‐8; LPS, lipopolysaccharide; TNF‐α, tumor necrosis factor‐α; ROR2, receptor tyrosine kinase‐like orphan receptor 2.

It is well known that pro‐inflammatory cytokines, such as TNF‐α, IL‐1β and IL‐8, are key mediators in LPS‐induced inflammatory responses. Effect of ROR2 downregulation on pro‐inflammatory cytokine release from LPS‐treated A549 cells in vitro is illustrated in Figure 6. In comparison with control group, LPS‐challenged A549 cells with or without negative control siRNA treatment released higher levels of TNF‐α, IL‐1β, and IL‐8. In contrast, the levels of TNF‐α, IL‐1β, and IL‐8 were significantly decreased by downregulation of ROR2 when compared with LPS treatment.

### 3.4 Downregulation of ROR2 inhibits JNK and ERK signaling pathway in LPS‐treated A549 cells in vitro

**Figure 7:**
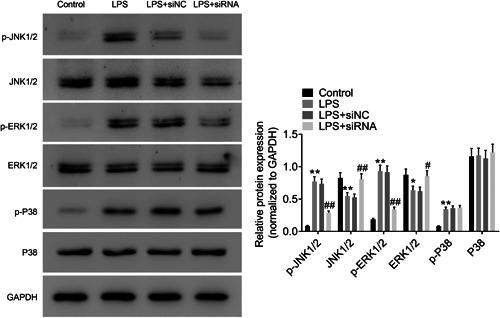
Effect of ROR2 downregulation on JNK and ERK signaling pathways. Values are expressed as mean ± standard deviation (*n* = 3). SPSS 18.0 was used for data analysis using Student's *t* test or ANOVA with the Dunnett's multiple comparison test. *p < .05, **p < .01 compared with control group; ^#^ *p* < .05, ^##^ *p* < .01 compared with LPS + siNC group. ANOVA, analysis of variance; ERK, extracellular signal‐regulated kinase; JNK, c‐Jun N‐terminal kinase; LPS, lipopolysaccharide; pJNK, phosphorylated c‐Jun N‐terminal kinase; ROR2, receptor tyrosine kinase‐like orphan receptor 2.

As shown in Figure 7, the phosphorylation of JNK, ERK1/2, and p38 was markedly increased after LPS administration in A549 cells when compared with control group. The phosphorylation levels of JNK and ERK1/2 were significantly decreased by ROR2 downregulation in comparison with LPS treatment. The findings suggest that downregulation of ROR2 inhibits JNK and ERK signaling pathways in A549 cells treated with LPS.

## 4 DISCUSSION

ALI is a common respiratory illness featured by severe hypoxemia, massive release of pro‐inflammatory cytokines, and alveolar epithelial cell damage.^15^, ^16^ Previous studies showed that inflammatory responses and pulmonary epithelial cell apoptosis were associated with the occurrence of ALI.^17^, ^18^ In accordance with the earlier reports, we found that evident pathological alterations were observed in the lung tissues of LPS‐challenged mice based on H&E staining results. In the current study, murine models of ALI were successfully established by intratracheal instillation of LPS. Furthermore, qPCR and immunohistochemical analysis revealed that the mRNA and protein expression of ROR2 were upregulated in the lung tissues of LPS‐challenged mice, suggesting that ROR2 is involved in the pathogenesis of ALI and may be a potential therapeutic target for ALI.

It was reported that ROR2 plays crucial roles in cell cycle, cell proliferation, and apoptosis by initiating signaling cascades.^19^, ^20^ To better understand the mechanism of ROR2 in the pathogenesis of ALI, we conducted cytological studies to explore the effects of ROR2 downregulation on LPS‐treated A549 cells in vitro. The cytological studies focused on the interrelation among ROR2 downregulation, cell proliferation, cell cycle process, cell apoptosis, and inflammatory responses. Cell proliferation assay by CCK‐8 as well as cell cycle and cell apoptosis analysis by flow cytometry showed that LPS administration significantly decreased cell proliferation, resulted in cell cycle arrest at G1 phage, and elevated apoptosis rate of A549 cells. It has been reported that TNF‐α, IL‐1β, and IL‐8 are important pro‐inflammatory mediators in inflammatory responses, moreover, excessive release of the pro‐inflammatory cytokines play crucial roles in the pathogenesis and development of ALI.^21^, ^22^, ^23^ CCNB1 and CDC2 are known to be important cell cycle regulatory proteins, and play key roles in promoting cell cycle progression.^24^, ^25^ Caspases, especially caspase‐3 and caspase‐9, are considered as crucial effectors of cell apoptosis.^26^ In the current study, we evaluated the effect of ROR2 downregulation on the expression of CDC2 and CCNB1, and cell apoptosis‐associated proteins, caspase‐3, and caspase‐9. Western blot results showed that ROR2 downregulation elevated the expression of CDC2 and CCNB1, and lowered the expression of caspase‐3 and caspase‐9 in LPS‐treated A549 cells, indicating that ROR2 downregulation alleviates cell cycle arrest and inhibits cell apoptosis. On the contrary, Cai et al. reported that MSCs that overexpress ROR2 attenuated LPS‐induced pulmonary inflammation.^27^ This discrepancy may be related to cellular microenvironment and intercellular interaction.

It is acknowledged that JNK and ERK1/2 are important proteins in regulating inflammatory responses and cell apoptosis.^28^, ^29^, ^30^ To preliminarily elucidate the mechanism that ROR2 downregulation alleviates inflammatory responses, cell cycle arrest, and cell apoptosis, we examined the JNK and ERK signaling transduction pathways. Western blot results showed that the phosphorylation levels of JNK and ERK1/2 were significantly decreased by downregulating ROR2 in comparison with LPS treatment, suggesting that ROR2 downregulation inhibits JNK and ERK signal pathway.

In conclusion, the current data indicate that ROR2 promotes LPS‐induced A549 cell injury at least partially through JNK and ERK signaling pathways. This novel mechanism provides novel potential therapeutic targets for ALI. However, the current research has some limitations: (1) the data in the present study were based on the A549 cell line, and there was a lack of primary lung endothelial cells; (2) we only focused on male mice, thus, the mechanistic and functional significance of ROR2 in female mice should be further verified; (3) the regulatory mechanism of ROR2 action also requires further investigation.

## AUTHOR CONTRIBUTIONS

Zhonglin Wang and Liu Yang conceived and designed the experiments. Zhonglin Wang and Liu Yang analyzed and interpreted the results of the experiments. Zhonglin Wang and Liu Yang performed the experiments; all authors have read and approved the manuscript.

## CONFLICT OF INTEREST STATEMENT

The authors declare no conflict of interest.

## ETHICS STATEMENT

The animal study protocol was approved by the Ethics Committee of Yongchuan Hospital Affiliated to Chongqing Medical University [2019 (204)].

## Data Availability

All data generated or analyzed during this study are included in this published article.
